# Supplementary figures and images for: Phosphoinositide-binding proteins mark, shape and functionally modulate highly-diverged endocytic compartments in the parasitic protist Giardia lamblia
Source: PLoS Pathog. 2020 Feb 24;16(2):e1008317. doi: 10.1371/journal.ppat.1008317 (PMC7058353; doi:10.1371/journal.ppat.1008317)

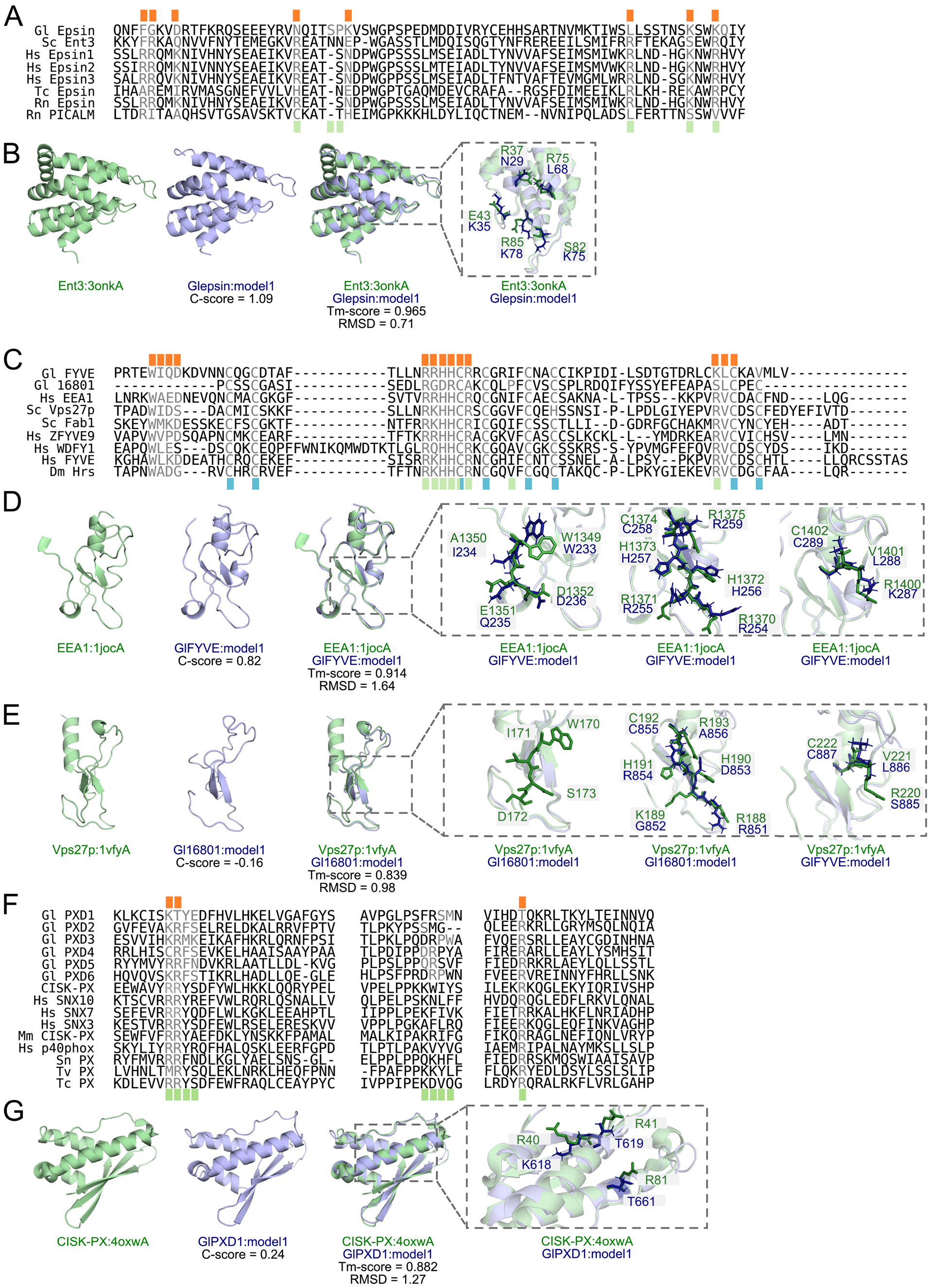

Supplement: S1 Fig — For all PIP-binding modules except GlNECAP1, the sequence of the lipid-binding domain was aligned to its respective homologous domains. Each domain was structurally modelled using I-TASSER (blue) and superimposed on its closest structural homolog (green). For each structural overlay a TM-score and RMSD value are reported, followed by a blow-up of the calculated location of known and predicted PIP-binding motifs. (A-B) Glepsin, (C-E) GlFYVE and Gl16801, (F-L) GlPXD1-6, (M-O) GlBAR1 and GlBAR2, (P-Q) GlFERM and (R-T) GlPROP1 and GlPROP2. (U) Legend to color code for conserved/similar residues. (V) Closest structural homologues, including their origin and identifiers, for structural overlay analysis of PIP-binding modules in G. lamblia. (W) Selected orthologues, including their origin and identifiers, for each G. lamblia PIP-binding module used in the MSA analysis to highlight conserved/similar residues for lipid-binding. (TIF) [file ppat.1008317.s001.tif]

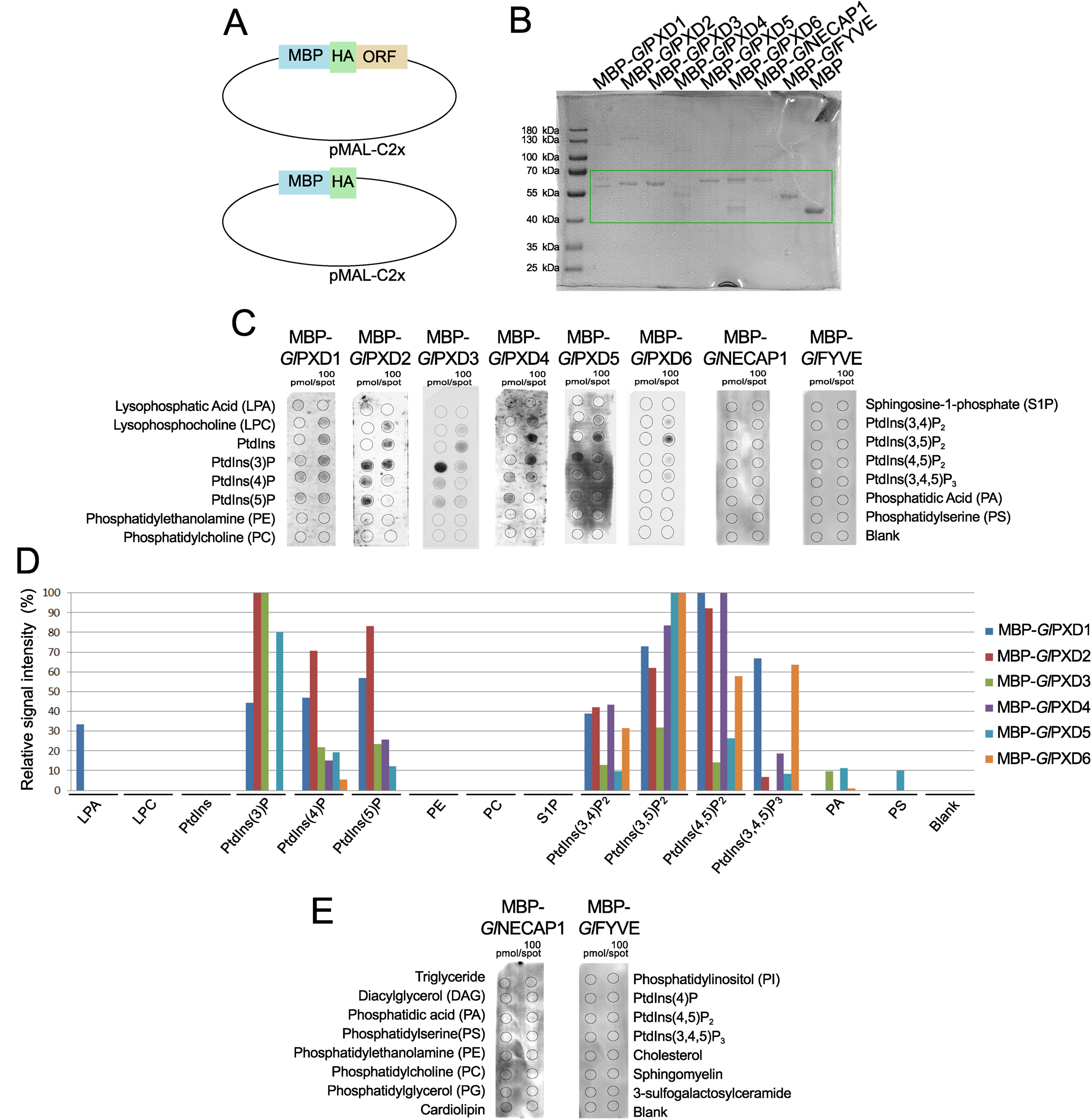

Supplement: S2 Fig — Lipid-binding and immuno-detection analysis of G. lamblia PIP-binding domains from proteins GlPXD1-6, GlFYVE and GlNECAP1 using lipid strips. (A) Schematic diagram of the pMAL-p2Cx vector used for heterologous expression of individual PIP-binding domains in E.coli. (B) SDS-PAGE analysis of recombinant epitope-tagged MBP-PIP binding domain fusions normalized to 1μg total protein. Protein ladder sizes are included in the first lane. (C) Immuno-detection of epitope-tagged MBP-fusions for each PIP-binding domain overlaid on lipid strips carrying spotted lipid residues at 100 pmol/spot and visualized by chemiluminescence. (D) Lipid binding preferences for all tested MBP-domain fusions, measured using FIJI and visualized as plots of relative signal intensity for each probed lipid residue. Values were normalized to those of lipid residues presenting strongest signal intensity. (E) Lipid binding preferences for GlNECAP1 and GlFYVE investigated using a different set of spotted lipid residues revealed GlNECAP1’s exclusive affinity for cardiolipin. (TIF) [file ppat.1008317.s002.tif]

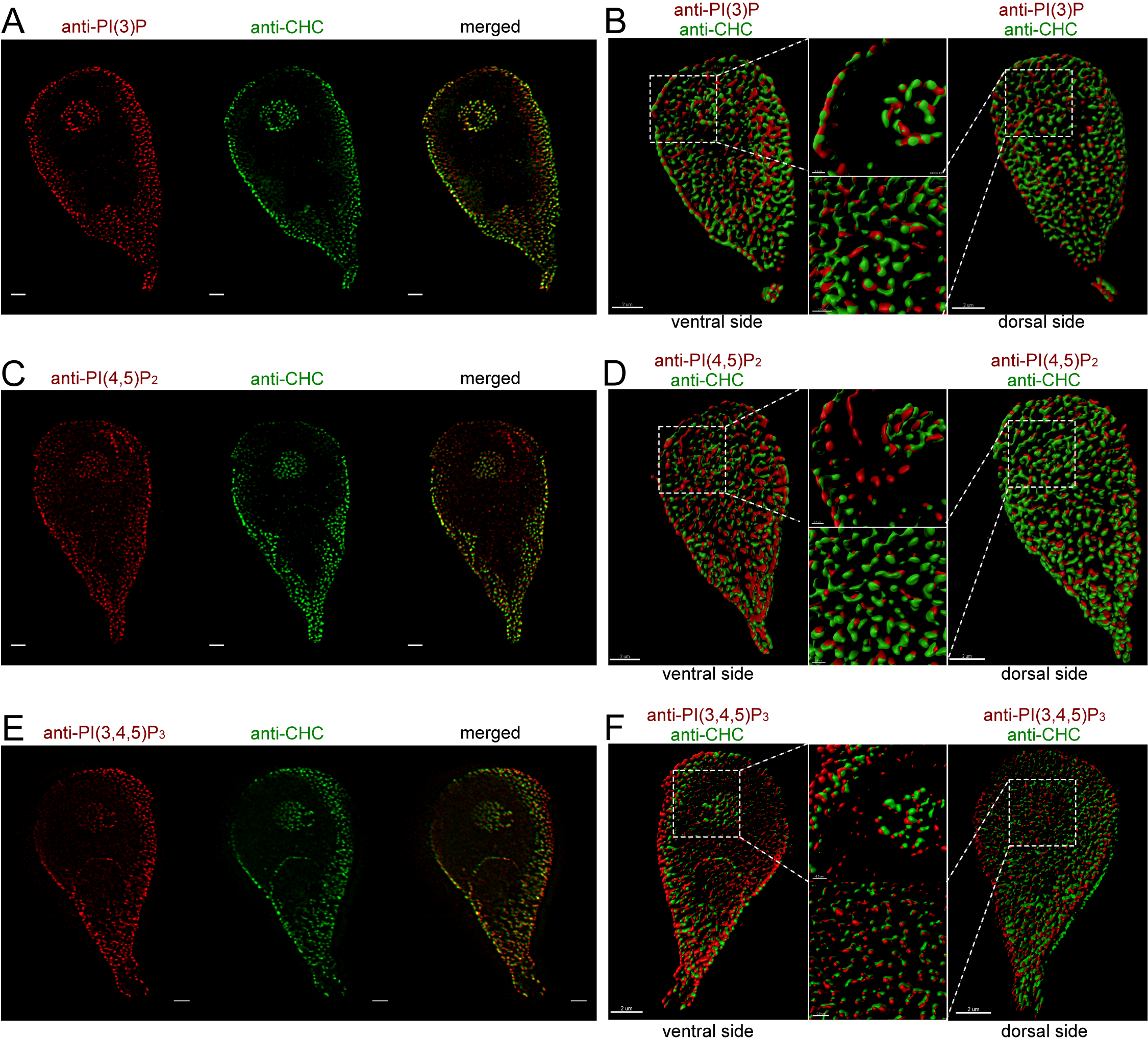

Supplement: S3 Fig — 3D STED microscopy analysis followed by signal overlap and deconvolution of representative non-transgenic wild-type G. lamblia trophozoites co-labeled with anti-GlCHC (in green) antibody and either (A-B) anti-PI(3)P, (C-D) anti-PI(4,5)P2, or (E-F) anti-PI(3,4,5)P3 antibodies (in red). Dorsal and ventral sides are defined with respect to the ventral disk. Scale bar for (A, C, E): 1 μm. Scale bar for (B, D, F): 2 μm. Scale bar for insets in (B, D, F): 0.5 μm. (TIF) [file ppat.1008317.s003.tif]

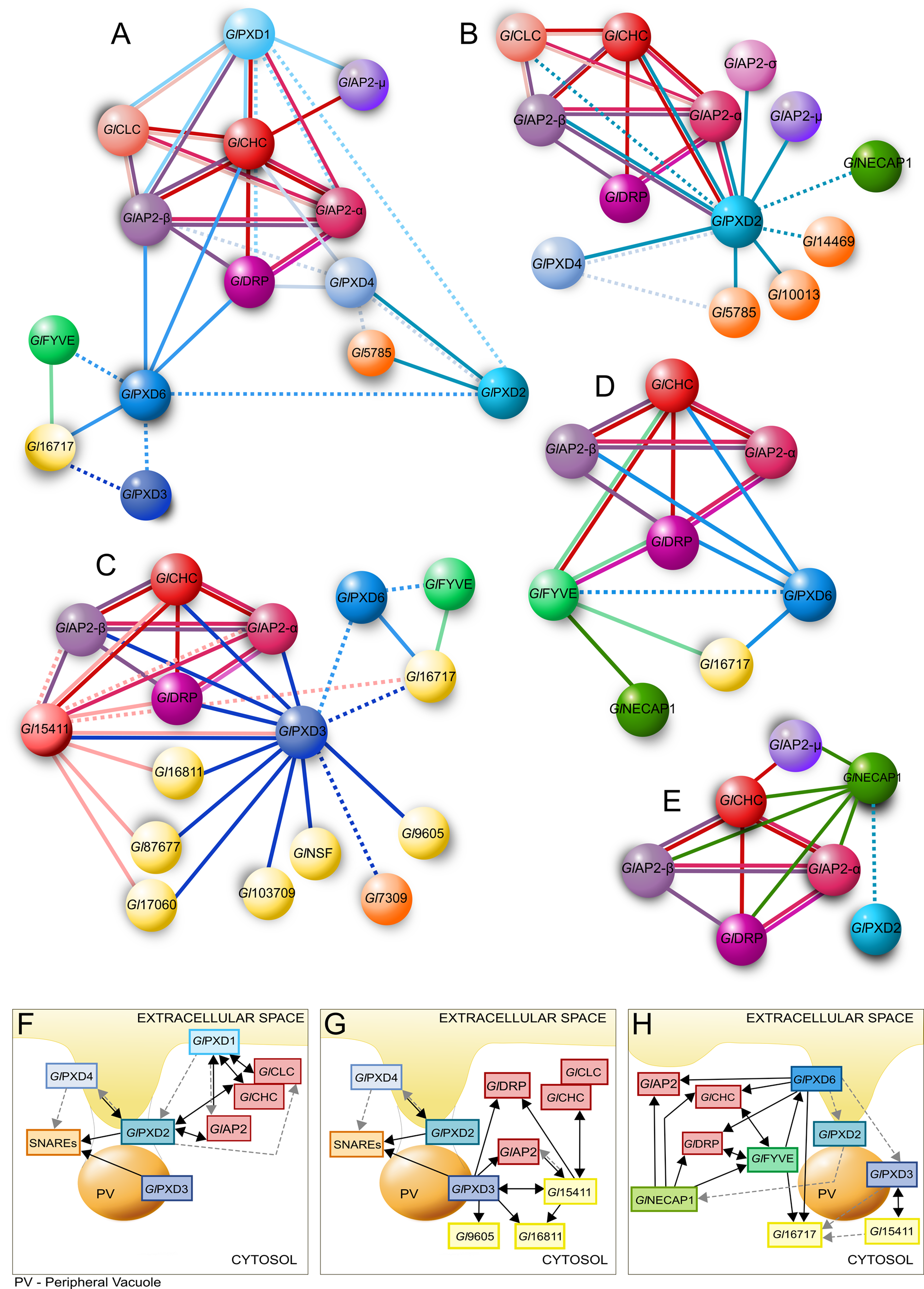

Supplement: S4 Fig — (A) Curated interactomes for GlPXD1, GlPXD4 and GlPXD6. All three epitope-tagged variants used as affinity handles in co-immunoprecipitation experiments identify GlCHC as a strong interaction partner for GlPXD1, 4 and 6. GlPXD1 and 4 further interact with other known clathrin assembly components such as GlCLC, GlAP2 subunits α, β and μ, and GlDRP. GlPXD2, albeit at low stringency, is the only other PXD protein found in all three interactomes. The GlPXD4 interactome includes a putative SNARE protein (5785; [45] while GlPXD6 as an affinity handle pulled down another PIP residue binder, GlFYVE, known to be associated to clathrin assemblies in G. lamblia [19]. (B) The curated extended interactome for GlPXD2 includes all core clathrin assembly components (GlCHC, GlCLC, all GlAP2 subunits, GlDRP [19]) and includes PIP-binders GlPXD4 and GlNECAP1. Three putative SNAREs Gl5785, Gl10013 and Gl14469 were also detected as bona fide GlPXD2 interaction partners, the latter previously detected in the GlPXD4 interactome. (C) Analysis of the extended GlPXD3 interactome using an epitope-tagged variant as affinity handle reveals robust interactions with clathrin assembly components GlCHC, α and β GlAP2 subunits, and GlDRP. Predicted inactive NEK kinase 15411 [48] is similarly associated to clathrin assemblies [19] and further shares proteins Gl16811, Gl87677 and Gl17060 as interaction partners with GlPXD3. Predicted SNARE protein Gl7309, GlNSF (GL50803_1154776) and proteins Gl103709 and Gl9605 are unique GlPXD3 interaction partners. The GlPXD3 interactome is connected to the GlPXD6 circuit both directly and through Gl16717. (D) The extended interactome analysis of epitope-tagged GlFYVE confirmed tight association to GlCHC, GlDRP and GlPXD6. GlNECAP1 as an alternative PIP-binding module was also detected. (E) A GlNECAP1-centered interactome highlights association to clathrin assembly components and to additional PIP-residue binders GlFYVE and GlPXD2. For all interactomes: solid lines: [file ppat.1008317.s004.tif]

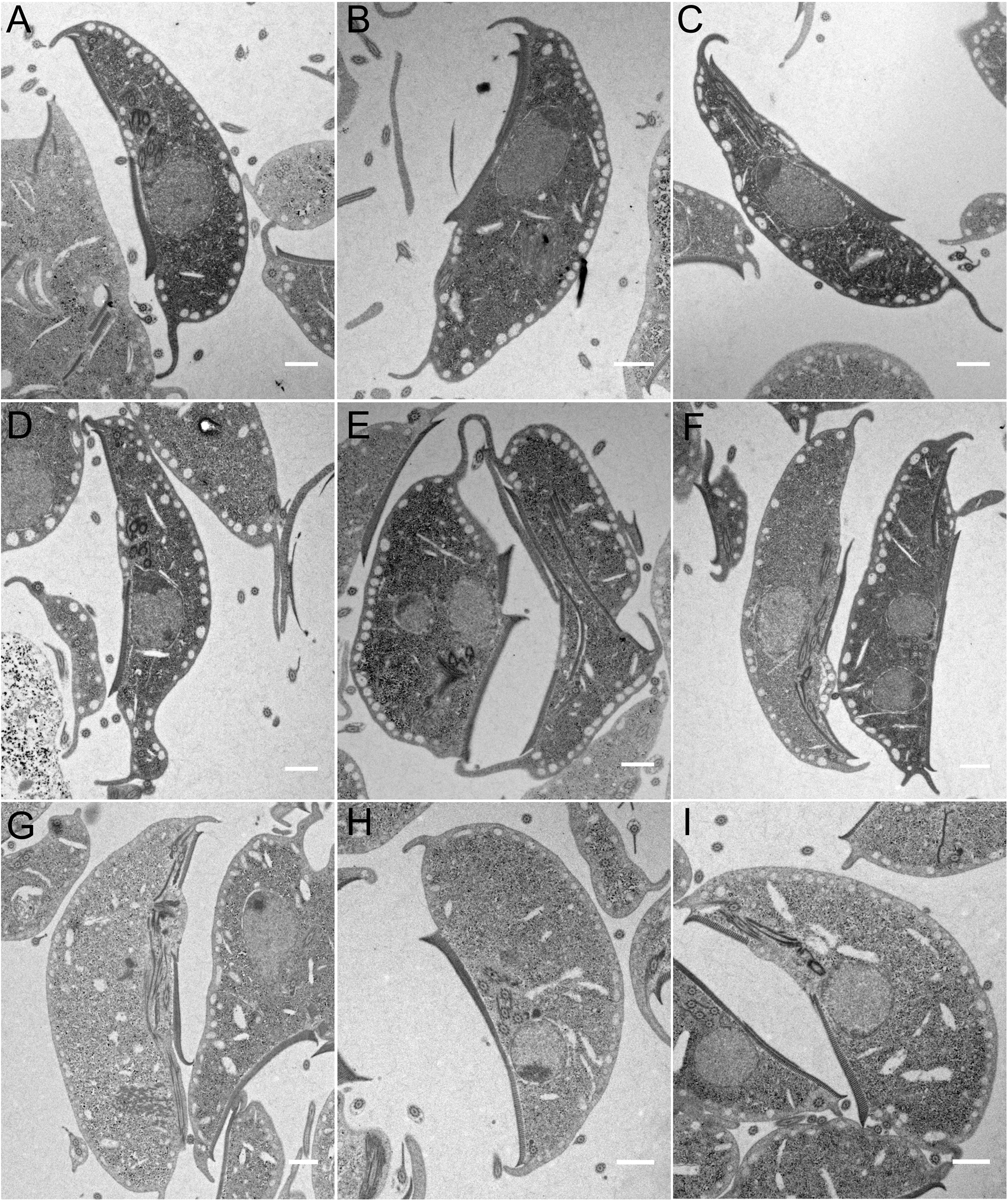

Supplement: S5 Fig — (A-F) Representative images of transgenic G. lamblia cells expressing construct pE::GlNECAP1-APEX2-2HA showing enlarged PVs and a diffused APEX-dependent cell staining signal. (G-I) Non-transgenic control cells. Scale bar: 1μm. (TIF) [file ppat.1008317.s005.tif]

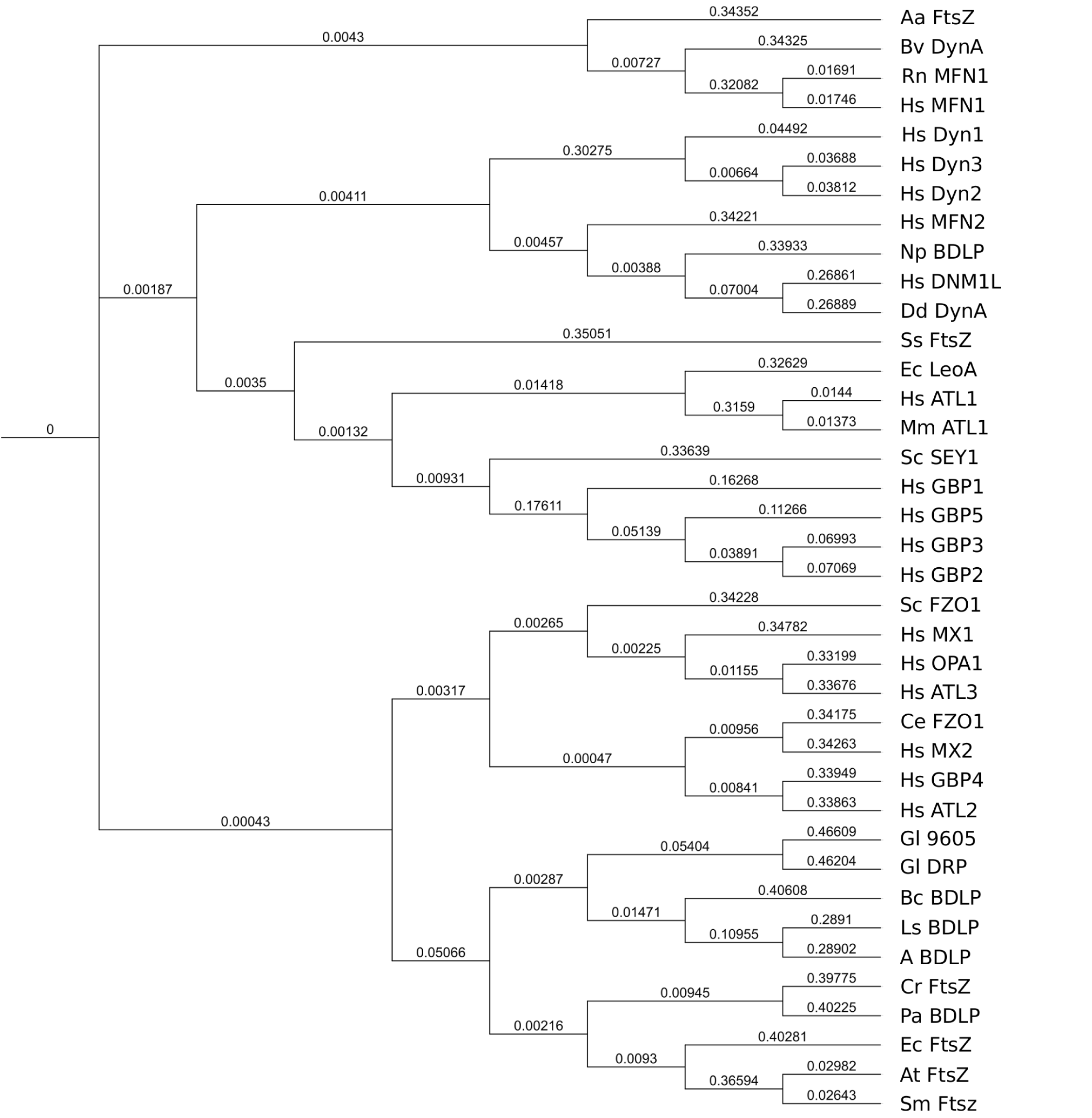

Supplement: S6 Fig — Phylogenetic analysis of predicted GTPase domains from the following prokaryotic and eukaryotic species used to compute the tree shown in the figure, including branch lengths as a measure of evolutionary distance: Aa—Aquifex aeolicus, Bv—Bacillus velezensis, Rn—Rattus norvegicus, Hm—Homo sapiens, Np—Nostoc punctiforme, Dd—Dictyostelium discoideum, Ss—Synechocystis sp., Ec—Escherichia coli, Mm—Mus musculus, Sc—Saccharomyces cerevisiae, Ce—Caenorhabditis elegans, Gl—Giardia lamblia, Bc—Bacillus cereus, Ls—Lysinibacillus saudimassiliensis, A—Anoxybacillus sp., Cr—Chlamydomonas reinhardtii, Pa—Pseudomonas aeruginosa, At—Agrobacterium tumefacies, Sm—Sinorhizobium meliloti. (TIF) [file ppat.1008317.s006.tif]
