# Supplementary material for: Phosphoinositide-binding proteins mark, shape and functionally modulate highly-diverged endocytic compartments in the parasitic protist Giardia lamblia
Source: PLoS Pathog. 2020 Feb 24;16(2):e1008317. doi: 10.1371/journal.ppat.1008317 (PMC7058353; doi:10.1371/journal.ppat.1008317)
Supplement: Attachment — Submitted filename: Cernikova et al_response to reviewers.docx [file ppat.1008317.s020.docx]

***Cernikova et al., Response to the Reviewers_*** ***manuscript PPATHOGENS-S-19-01940***

***Reviewer #1:*** Cernikova et al have addressed a longstanding question about the identity of PIP binding proteins in Giardia and their role in regulating endocytic trafficking. They used sensitive bioinformatics tools to identify candidate PIP binding proteins, modeled these proteins to test whether they had conserved residues involved in PIP and lipid binding, and then tested lipid binding activity with in vitro assays. They characterized the localization of these proteins and their relationship to clathrin and fluid phase endocytic cargo using STED super-resolution imaging. Further they explored the function of these proteins in Giardia using a combination of approaches including saturation of binding sites with over expressed PIP binding proteins, dominant negative mutants and chemical inhibitors. They performed crosslinking and pulldown experiments to identify the interactome of several validated lipid binding proteins and established that many of these proteins are in the same clathrin interaction network. They also performed EM demonstrating that some of the dominant negative proteins caused defects in PV morphology. Overall the paper is a major advance and represents a substantial amount of work.

*>> We thank the reviewer for her/his appreciation of our work.*

What detracts from the work is a focused concise message and a model for how these perturbations block endocytosis.

*>> We have included a model in the first version of figure 10 where we summarise PIP-related interactomes with respect to clathrin heavy chain, zooming into specific interactomes. However, we still do not fully understand how these networks function and how perturbations in the system arise. Therefore, based on the available data we feel it is premature to make more definitive statements on how and why the endocytic system is affected by e.g. over-expression of an individual factor.*

In my opinion there is simply too much data packed into the main figures which will make tracking key points a challenge for the general readership of Plos Pathogens.

While the manuscript initially focuses on PIP binding proteins the proteomics results lead to tangents that I think could be reserved for future studies.

*>> We take this point and we have restructured our manuscript to prune side-stories that detract from the core work. Please see our detailed interventions below.*

Rather than following up on new proteins pairwise co-localization studies of the identified lipid binding proteins would lead to a more comprehensive understanding of how these proteins are organized in relationship to each other.

*>> We have already partially addressed this issue by showing that PIP-binding proteins can be reciprocally co-immunoprecipitated and that they very often reside in the same subcellular compartment, indicating how they are likely to participate in the same pathway and/or complex. Nevertheless, not to detract from our main work, we have made an effort to prune side-stories (see comment above).*

***Reviewer #2:*** Cernikova et al have studied the importance of PIPs in Giardia endocytosis. It is overall a well-performed study but some of studies have been performed earlier and the outline of the paper can be changed to make it more accessible.

*>> Although we have referenced former work from other groups, we have revised our manuscript to include more references to past publications, specifically Cox et al., 2006 and Hernandez et al., 2007. We have significantly re-structured our manuscript for better accessibility, as suggested by reviewer 2. Please see our detailed interventions below.*

***Reviewer #1:*** Major Issues:

1. Figure 2 The presented gradients blots seem inconsistent with the strip blots found in the supplement. For example PXD3 very clearly has a dominant preference for PI(3)P on the strip blot but this is less clear on the gradient blot. The number of replicates is not indicated.

*>> We performed three experiments using strips and arrays. Given that both lipid strips and arrays are known to vary even between units of the same batch, we underline that any data generated using these tools should act as lead to more highly controlled experiments on lipid binding preference.*

*The variation in binding preference for e.g. PXD3 can be explained by the fact that usage of lipid strips as opposed to lipid arrays will lead to different binding dynamics. Lipid strips carry fixed amounts (100 pmol) of very diverse lipid residues whereas arrays are spotted with gradients (100, 50, 25, 12.5, 6.25, 3.13, and 1.56 pmol) of fairly similar yet specific PIP residues. We used both strips and arrays to address two different questions i.e.: a) which kind of lipid residue is bound preferentially by a given PIP-binding domain?; b) within the PIP family of lipid residues, which subtype is bound preferentially by a given PIP-binding domain? Lipid strips and arrays were used to address questions (a) and (b), respectively.*

2. Another issue is that the methods indicates that the MBP fusions were lyophilized. Some proteins cannot tolerate lyophilization as this treatment could impact folding and activity. So were the proteins tested for activity before lyophilization? Of particular interest are the proteins that showed no binding activity. If activity was only tested after lyophilization, then the authors should repeat the lipid binding assays with proteins tested immediately after purification.

*>> We thank the reviewer for pointing this out because it highlights a gap in how we detailed our protocols. For the arrays, we tested proteins that were NOT lyophilized, rather snap-frozen in liquid nitrogen immediately after purification, thereby minimizing artefacts. We did make use of lyophilization for the lipid strip-based experiments. We have added this missing information in the revised manuscript. However, we’d like to underline that recombinant GlNECAP1 protein’s affinity to cardiolipin could only be determined using lipid strips and that this specific binding preference was recorded using both lyophilized AND snap-frozen protein sample, suggesting lyophilization likely did not severely impact the protein’s binding capacity. For this reason, we stand by our decision to include the lipid strip-derived data in supplementary figure 2 and main figure 2, specifically for GlNECAP1.*

3. The authors indicate that their structure prediction identified key preserved residues. If they would mutate some of these for example in the most divergent of the identified proteins with lipid binding activity, this would help support the accuracy of the structure prediction. This could be performed in strip blots or through localization as one would predict the proteins of interest would no longer localize to PVs or bind the blotted lipids.

*>> This is a good suggestion to be followed up in a targeted and full characterization of PIP-binding proteins in G. lamblia, which, at this point, is beyond the immediate scope of our current manuscript and would run contrary to the recommendation of both reviewers to streamline the manuscript.*

4. Figure 3. 2mM neomycin seems like a lot of antibiotic and the duration of treatment was not specified. I understand Giardia should not be sensitive to this antibiotic, but I wonder if the authors checked viability of the cells before the endocytosis assay.

*>> During revision, we realized we had miscalculated the amount of neomycin used for this assay which was 7.2mM. 2mM neomycin is the concentration generally used to select for transfectants over a 4-5 day period (Sun et al., 1998; Su et al., 2007; Wampfler et al., 2014; Ebneter et al., 2016). We have corrected the concentration and specified the duration of treatment in the revised manuscript. Based on the maintenance of cell integrity as shown by the DIC microscopy images, the length of treatment was not sufficient to impact their viability. To address the reviewer’s question, we imaged living cells after treatment with either no, 2mM, 7.2mM and 15mM neomycin at 37°C for 45 minutes. Time-lapse imaging shows that motility was not affected, suggesting that cells were vital. These videos have now been included as supplementary data.*

*As shown and discussed in Mayer et al., 2000 and Gabev et al., 1989, the concentrations of neomycin needed to affect PI(4,5)P2 turnover in different systems are quite variable, ranging from 10 μM to 10 mM. Given that this treatment had never been tested on Giardia before to elicit PIP-related short-term phenotypes, we opted for a higher concentration.*

Also there are two publications that performed bioinformatics to assess phosphoinositide kinase signaling in Giardia and showed that LY29004, an inhibitor of phosphoinositide kinases, impacts cell growth (Cox 2006 and Hernandez 2007). Rather than trying to saturate PIP sites their existence can be blocked using wortmanin or LY294002. One would predict that this would result in altered protein localization and ESV morphology.

*>> We decided to make use of neomycin because several reports underline its mode of action as a highly specific PI(4,5)P2 binder, with reduced affinity for PI(3,4,5)P3 (Schacht, 1976; Gabev et al., 1989; Arbuzova et al., 2000). Since we hypothesized that the biological availability of PIP residues could impact fluid-phase uptake (as measured by Dextran uptake) and possibly PV function, we chose neomycin due to its marked binding preference for PI(4,5)P2, a PIP residue known to play important roles in the regulation of endocytosis. Wortmannin and LY294002 are both PI3K inhibitors which therefore act downstream of PI(4,5)P2 and could generate a complex phenotype that could be challenging to dissect. Both molecules were in fact shown to inhibit cell growth (Hernandez et al., 2007).*

Additionally, the authors measured the cell width then did a t-test on the median cell size. They should either do a Mann-Whitney U on the median or they should use a t-test to compare means. (Mann-Whitney U is probably better.)

*>>* *As suggested by the reviewer, we performed a Mann-Whitney test on our data and could confirm the significance of the observed changes. Specifically: (i) WB vs FYVE p(0.05)-value = 2.22045e-16, therefore H0 is rejected, H1 is valid and transgenic GlFYVE-expressing cells are significantly larger than non-transgenic WB cells. (ii) WB vs P4C p(0.05)-value = 0.182377, therefore H0 is accepted and no significant change is measured. These data are now included in the revised version of the manuscript.*

5. Despite extensive super-resolution and EM imaging the authors do not propose any explanation for how their manipulations block endocytosis. Is it that the PVs are no longer open to uptake dextran or is it that they no longer can close so the dextran diffuses away during fixation?

*>> This is a challenging question to address because, to our knowledge, there is no subcellular high resolution microscopy data on individual PV dynamics in living cells. This means we are only able to observe and analyze the end result of our manipulations on an array of organelles. Indeed, our data shows the elicitation of uptake phenotypes upon perturbation of either PIP or PIP-binding protein homeostasis. For example, in figure 8 we present light and electron microscopy data on ectopic expression of GlNECAP1-derived constructs which show how perturbation of this PIP binder changes PV size and morphology. However, our EM images highlight the presence of closed and well-separated PV compartments, suggesting the defect does not lie in faulty closing of the organelles. On the contrary, we rather favour the hypothesis that uptake is affected i.e. linked to the ability of the PV compartments to fuse with the PM. This may not be the case for all the phenotypes we generated but we currently do not have the tools to address this question in more detail. We did however include this point in our discussion in the revised manuscript.*

6. Figure 7. How do the authors know that the NT-FYVE is generating a new compartment versus just aggregating? Also I don’t know what relevance this has to the biology of Giardia it seems like an artifact.

*>> In general, overexpression of GlFYVE and its truncated versions elicited severe dextran uptake phenotypes. All GlFYVE versions appear to associate with GlCHC. Only in the case of NT-FYVE expression do we see structures that are positive for the epitope-tagged construct and partially overlap with GlCHC labelling however, they appear to be unrelated to PVs (based on dextran labelling) and ESVs (based on CWP1 labelling). We agree with the reviewer that these may be aggregates and not compartments and we have amended our text to reflect this possibility.*

7. I have concerns about using the CWP1 promoter to drive over expression. Encystation is promoted by changes in cholesterol and pH which is accompanied by morphological changes that could impact PV function. The authors should, perhaps for their favorite protein, test whether they get similar phenotypes with the tet promoter system. Also it is surprising that the authors did not attempt to use any reverse genetics tools to assess the function of these proteins. Dominant negative and over expression studies can indicate a role but they can also produce misleading results whereas reverse genetics will cleanly indicate if a protein is required for a process of interest.

*>> Cells were subjected to a 6hr encystation pulse to test expression of GlFYVE and derived constructs and were always accompanied by non-transgenic controls similarly treated to test for dextran uptake efficiency. Encysting cells appeared to take up dextran as efficiently as non-encysting cells, suggesting that encystation had not impacted this function, at least not visibly in our conditions. However, we’d like to thank the reviewer for raising this issue because we realized our legend was not clear enough in stating our experimental conditions and we have amended it accordingly.*

*We use the pCWP1-based system for conditional expression of genes of interest because it is tightly regulated (essentially on/off), can be modulated easily, and relies exclusively on Giardia-specific sequences. Over the past 20 years we and others have implemented CWP controlled ectopic expression for a large range of genes and specifically designed variants including dominant-negative factors. Conversely, the Tet system, although of limited usefulness for some special applications, is by design not particularly suitable for Giardia because of the virtual absence of 5’ untranslated sequences and presents variables that have to do with TetR and TetO specificity which could be confounding factors for subsequent analyses. Yields of conditionally expressed proteins are very low and there is considerable leakiness of the system which constitutes a strong confusing factor when using this system to express e.g. dominant-negative variants of proteins ectopically expressed. For this reason, we preferred to use the pCWP1 system.*

*We agree with the reviewer that testing function using genetic tools is an ideal approach to a full functional characterization. However, the only report on a clean knock-out line in Giardia (Ebneter et al., 2016) demonstrates how laborious this year-long approach is. Recent reports concerning variants of CRISPR-Cas9 (McInally et al., 2019; Lin et al., 2019) are very encouraging. However, given they are not equivalent to a clean knock-out, show only partial penetrance and/or compensatory responses, and produce effects that are in most cases difficult or even impossible to interpret. Hence, we decided not to pursue these methods until they have been shown to be more robust and reliable.*

***Reviewer #1:*** Minor Issues:

1. Abstract, Author summary and Intro should start by saying “In model eukaryotes” to make it clear that the biology of Giardia is not being discussed.

*>> Addressed.*

2. The authors indicate that they could not identify C2 domain containing proteins yet a GiardiaDB text search of C2 yields four proteins. Some of which are phosphoinositide kinases which would be expected to bind PIPs? See GL50803_16728, GL50803_114201, GL50803_17406, GL50803_14855.

*>> The PIP-binding domains used as query sequences were mainly extracted from Lemmon et al., 2008. The 1CZS sequence was chosen to search for C2 domains which explains why we missed the ORF numbers listed by the reviewer. We have included this information to underline how our approach may lead to an underestimation of detected PIP-binding modules but also to highlight how sequence divergence plays an important role in determining detection limits.*

3. Figure 1 Panel B can be moved to the supplement as the relevant proteins are shown again in C-I. This also raises the point that the legend stops at C. For panel A, the current size of the DIC insets are too small to see anything, these could be expanded if the panel is moved into the supplement.

*>> We have restructured our figure to reduce redundancy. For this purpose, panel B now includes only images for the dextranOG control, Glepsin, Gl16801, GlPXD5, GlBAR1, GlBAR2, GlPROP1, GlPROP2 and GlFERM and the DIC insets have now been included as self-standing images. We removed panels corresponding to the same lines imaged in sections C to I. The removed panels have been eliminated to reduce redundancy.*

4. Several proteomics tables include cytoplasmic HSP70 but the text indicates that no clathrin uncoating proteins were found. Is cytoplasmic HSP70 not involved in clathrin uncoating?

*>> Cytoplasmic Hsp70 is not involved in CCV uncoating. Rather, it is a related protein called Hsc70 which has been shown to participate in CCV disassembly (Morgan et al., 2001; Boecking et al., 2018). The Hsp70 the reviewer refers to is an abundant cytosolic protein which is likely a contaminant. There are no reports on Hsp70 compensating for a missing Hsc70, Hsc70 being a CCV-specialized accessory protein.*

5. Figure 5. While the fact that snare proteins are part of the PXD2 interactome is intriguing, I think including the localization in the main text detracts from a focused story. I recommend removal of panel B. Additionally I think all the interactome data should be combined into one figure to reduce the total number of figures.

*>> We agree that this manuscript presents large amounts of data and that it could profit from a little pruning so we have eliminated section B from figure 5 and have relabeled the remaining sections accordingly. We did include all interactomes in the first version of supplementary Fig5.*

6. I am unaware of any particular genetics nomenclature for Giardia, but the authors use of :: to indicate protein fusions is confusing as it is more commonly used to indicate promoter fusions. In particular I was confused by pEHA::GlPXD2 it took me a long time to figure out this is the endogenous promoter driving an N-terminal HA fusion to PXD2. Perhaps pE::HA-GlPXD2 or pE::HA:GlPXD2 would be easier to understand.

*>> We agree with the reviewer that the nomenclature needs improvement and we settled for e.g. pE::HA-GlPXD2.*

7. Figure 6. I think the identification of another dynamin homolog in Giardia is fascinating, but I think its inclusion distracts from a focused story. especially since there is no discussion of how mechanistically the mutant blocks endocytosis. I think a detailed study of this new dynamin and the more conventional dynamin is totally warranted and could be packaged as a focused study with EM work to examine how these proteins are preventing endocytosis. On the other hand if the authors were to perform EM to examine whether the mutants alter ESV morphology this could help explain the endocytosis defect.

*>> We agree with the reviewer that having found a novel dynamin is of interest and we think it is valuable to report on the effect that mutating Gl9605 appears to have on uptake. This is especially important given that GlDRP has been previously implicated in PV functionality and morphology (Gaechter et al., 2008) and that no other DLP has been identified so far in G. lamblia. For this reason, we stand by our decision to include these data in this manuscript in their current shape, having laid the necessary groundwork for characterizations.*

8. For the proteomics analysis the methods do not indicate whether individual protein bands, entire lanes, or a certain number of segments were cut out for mass spec analysis?

*>> We clarified this in the materials and methods section in the revised version of the manuscript.*

9. Figure 8 legend. It isn’t clear which promoter was used to drive expression. The authors seem to use ectopic and overexpression interchangeably. I would consider ectopic to be on a plasmid not integrated into the genome and over expression to be when they use the CWP1 promoter to purposely drive expression beyond normal levels. The work would be easier to follow if this was clarified. Is 8D actually using the endogenous promoter as indicated in the graph?

*>> In figure 8, we present data derived from expressing GlNECAP1 under the control of its own putative promoter. We have revised our text to distinguish between these two expression conditions.*

10. Figure 9F dorsal view appears to be in a different orientation than the ventral view?

*>> This is due to the positioning of the cell and the fact that we imaged its dorsal and ventral sides separately.*

11. Figure 10. It would be helpful to include a key for the cartoon. Also the authors switched colors for dextran and HA in the middle of the paper. I recommend they always false color HA red and dextran green to match the model regardless of which fluorophores they used. The labels can simply say HA and Dextran and it will help the reader follow the work. 10D-G. In the interest of streamlining the paper I feel that one simplified overall interactome in the working model would be sufficient. The others could be in supplement.

*>> We have restructured this figure according to the reviewer’s suggestions. Specifically:*

*a)	we added “PV” to label the organelle in the figure*

*b)	we moved sections D, E and F to supplementary figure 4 which includes all sub-interactomes, and have amended the text accordingly.*

*c)	we have neutralized color in Fig10D since there is no specific code. The same was done for the removed panels now included in the revised FigS4.*

*However, we stand by our decision not to standardize HA and dextran labelling across the entire paper. There is a sound technical justification which is that only dextran-OG (and not dextran-TxR) can be used for STED microscopy with our specific antibody combination. In fact, the only time we’ve had to switch colours was when we reported on STED microscopy data. To underline this higher resolution imaging approach, we’d prefer to keep labelling color as it is currently.*

***Reviewer #2:***

1. Line 44, 55 proteins in what organism(s). In all eukaryotes?

*>> Addressed in the revised manuscript.*

2. Lines 94 and 95. There are of course also amino acid, sugar and nucleoside transporters in the membrane that are crucial for nutrient uptake.

*>> We agree with the reviewer that nutrient uptake requires a number of transporters. However, given how uptake occurs, the membrane involved is most likely to be the PV one and not the plasma membrane which is covered by a dense coat of VSPs. For example, in Banerjee et al., 2008, the authors report on a PV localization of the UDP-Glc transporter. For this reason, we still consider the current sentence as correct.*

3. I lack more references to already published studies of Giardia PIP binding proteins in the introduction.

*>>Although we have referenced former work from other groups, we have revised our manuscript to include more references to past publications, specifically Cox et al., 2006 and Hernandez et al., 2007.*

4. Line 124. In silico annotation techniques, what specifically is this?

*>> To clarify, we made use of in silico analysis and prediction tools including primary and secondary amino acid sequence analysis, functional domain prediction, ab initio structural modelling etc. These are listed in materials and methods.*

5. Line 132. Not all PIP interacting proteins are associated with endocytosis.

*>> We agree with the reviewer and we have not written otherwise. In line 132 of the submitted manuscript, we clearly state that our interest is in endocytosis-related PIP binding modules and we searched the genome for homologues of 14 PIP binding modules, only some of which are endocytosis-associated.*

6. Line 175. It is good to make it clear that the localizations were done in this study.

*>> We stated this in lines 178 to 183 of the original manuscript.*

7. Line 192. It is not clear to me why these particular proteins were selected for further studies.

*>> Our focus for this manuscript is PV-mediated fluid-phase uptake. Given that we have previously investigated the GlCHC interactome to dissect endocytosis-related components (Zumthor et al., 2016) and given that PVs are responsible for uptake, we selected PIP-binding domains that had been previously reported to be connected to clathrin assemblies. We included GlPXD3-6 since, along with GlPXD1 and 2, they constitute a family of PXD proteins.*

8. Line 197. Was overexpression with another promoter tested on the PXD5 protein to see a stronger signal? I see a signal in 1B.

*>> There is indeed a faint signal for GlPXD5 in section B. However, we only tested expression using a putative endogenous promoter for this construct. This proved to be insufficient for further investigations such as co-immunoprecipitation and high-resolution STED microscopy.*

9. Line 199. G. muris data is now available at NCBI, are all the proteins conserved there?

*>> We have performed this search as suggested by the reviewer and the data is summarized in the table below. We could find well-conserved albeit scarcely annotated homologues for all selected PIP-binders associated to endocytosis in G. lamblia.*

10. Line 210. This has already been done in an earlier study by Jana et al, 2017.

*>> We have added the missing reference to our revised text.*

11. Line 230. Compare new data with old data from Jana. Add the PIP binding data to Table 1.

*>> Table 1 includes references to past reports that have investigated PIP binding proteins in Giardia and their binding preferences. We have correctly cited both Jana et al., 2017 and Sinha et al., 2011. We have amended table 1 by including an additional column on binding preferences reported in these previous publications. Our own data on PIP binding preferences only comes in later, in figure 2.*

12. Line 245. How do the authors now that it is saturation, was a titration curve done?

*>> We did not perform a titration curve. In line with the reviewer’s question, we have amended our text in the revised version to reflect perturbation and reduced availability of free binding sites for the specific PIPs we targeted.*

13. Line 251. The controls could have been a parasite overexpressing another PIP domain containing protein not associated with the PVs.

*>> We agree with the reviewer that these could have also been viable controls. However, given that we do not know what the interactomes of PIP-binding proteins not associated to PV function are, we would run the risk of eliciting phenotypes which could confound our analysis. Since PIP binders have affinity for molecules that often play important regulatory roles in cell function and physiology, using non-transgenic (wild type) cells is the most conservative and parsimonious approach to controlling for transgene-induced phenotypes.*

14. Line 264, Neomycin kills Giardia, can this affect uptake?

*>> During revision, we realized we had miscalculated the amount of neomycin used for this assay which was 7.2mM. This mistake was corrected in the revised manuscript. Giardia is sensitive to this antibiotic at 2mM, which is the concentration generally used to select for transfectants over a 4-5 day period (Sun et al., 1998; Su et al., 2007; Wampfler et al., 2014; Ebneter et al., 2016). However, based on the maintenance of cell integrity as shown by the DIC microscopy images, the length of treatment was not sufficient to impact their viability. To address the reviewer’s question, we imaged living cells after treatment with either no, 2mM, 7.2mM and 15mM neomycin at 37°C for 45 minutes. Time-lapse imaging shows that motility was not affected, suggesting that cells were vital. These videos have now been included as supplementary data.*

15. Line 286. The whole interactome part to line 423 can be condensed and the results presented in one table. The follow up data specific for each protein can complement this

*>> We have condensed the text and included a new table 2, in line with both reviewers’ suggestions.*
